# Protocol for real-time measurement of mitochondrial bioenergetics in 3D-cultured brain tumor stem cells using the Resipher system

**DOI:** 10.1016/j.xpro.2025.103651

**Published:** 2025-02-24

**Authors:** Cloé Tessier, Maxime Toujas, Antonio C. Pagano Zottola, Andreas Bikfalvi, Thomas Mathivet, Thomas Daubon, Lucie Brisson, Audrey Burban, Ahmad Sharanek

**Affiliations:** 1 University of Bordeaux, INSERM UMR1312, BRIC BoRdeaux Institute of Oncology, Bordeaux, France; 2 University of Bordeaux, CNRS, IBGC, UMR5095, Bordeaux, France

**Keywords:** Cell-based Assays, Cancer, Metabolism

## Abstract

Aberrant mitochondrial function can lead to severe human diseases, including neurodegenerative diseases and cancer. Here, we describe a cell-based protocol for measuring different mitochondrial respiratory parameters using the high-resolution real-time Resipher system. We optimized this protocol on brain tumor stem cells cultured in three-dimensional spheroids. We provide essential optimization steps for cell seeding density, mitochondrial respiration modulator concentrations, running the assay, and data analysis.

For complete details on the use and execution of this protocol, please refer to Burban et al.^1^

## Highlights

• Protocol for real-time oxygen consumption measurement in 3D stem cells using Resipher
• Determining optimal cell density to ensure accurate oxygen consumption measurements
• Procedure to identify the ideal concentration of mitochondrial respiration modulators
• Technique to analyze different mitochondrial respiration parameters with modulators

## Abstract (editor highlights)

Publisher’s note: Undertaking any experimental protocol requires adherence to local institutional guidelines for laboratory safety and ethics.

## Graphical abstract

**Figure:**
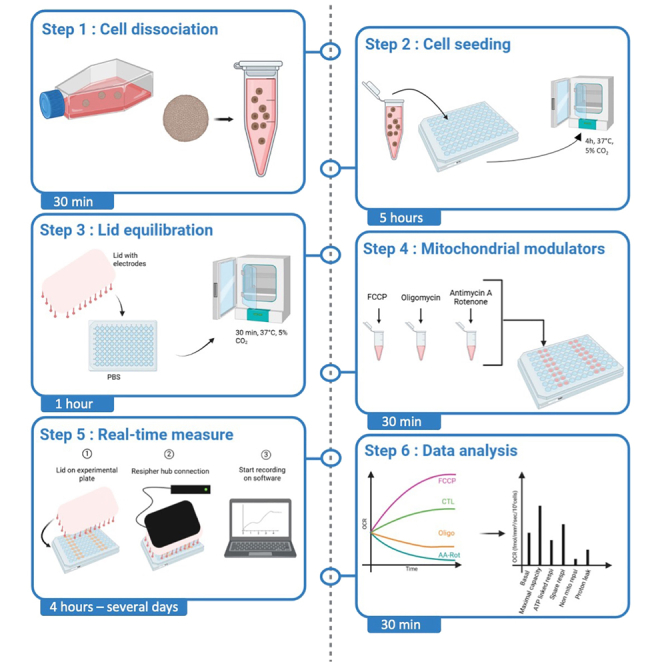

## Before you begin

Mitochondria are the powerhouses of the cell and produce chemical energy in the form of adenosine triphosphate (ATP) in a process termed oxidative phosphorylation (OXPHOS). In addition to their role in energy production, they have critical functions in intracellular calcium homeostasis, steroid metabolism, fatty acid β-oxidation, apoptosis, redox balance, and the production of biosynthetic molecules. Because of the multifaceted contribution of mitochondria to key biological and metabolic pathways, it is not surprising that mitochondrial dysfunctions can have detrimental consequences and are identified in many common pathologies, including cardiovascular diseases, neurodegeneration, aging, metabolic syndrome, and cancer.^2^

Mitochondria harbor the electron transport chain (ETC), the machinery required to perform OXPHOS. The ETC consists of complexes I–V and couples electron transport to the synthesis of ATP in the mitochondrial matrix.^3^ Since oxygen is consumed mainly in the process of ATP production by mitochondrial OXPHOS, the oxygen consumption rate (OCR) represents an integrative and comprehensive readout of mitochondrial function.^4^ Although complex IV is the only oxygen consumer, addition of pharmacological modulators allows the assessment of different respiratory parameters, including total cellular respiration, non-mitochondrial respiration, maximal respiration, spare respiratory capacity, ATP-linked respiration and proton leak.^5^

Several methods are available for assessing OCR, including but not limited to the Seahorse XF Flux Analyzer, Oroboros 2k respirometry systems, and the recently developed Resipher system by Lucid Scientific. Unlike traditional, large OCR analytical instruments, the Resipher system is a hand-held, user-friendly device that enables real-time monitoring of oxygen consumption directly within standard cell culture plates and incubators. The advantages and limitations of these methods are summarized in Table 1. A key advantage of the Resipher system is that it is an open system capable of measuring the oxygen concentration gradient in the media above cells under standard culture conditions. This enables the real-time monitoring of OCR changes over several weeks. Additionally, unlike the Seahorse system, which requires the replacement of culture media with Seahorse XF assay media, the Resipher system uses standard culture media, thereby avoiding potential nonspecific effects on respiration that may result from media replacement. Similar to other methods for measuring OCR, the Resipher system requires optimization for each cellular model system, depending on its mitochondrial respiration capacity and sensitivity/responsiveness to different respiration modulators. Although protocols for techniques such as Seahorse and Oroboros are well-documented, only two very recent protocols describe the Resipher technique adapted for 2D cell cultures.^6^^,^^7^ To the best of our knowledge, our protocol is the first to provide an optimized method for using the Resipher system in 3D cultures, as no such protocol has been described previously.

**Table 1:** Comparison of existing methods to measure oxygen consumption

	Advantages	Disadvantages
Resipher system	• Screening of multiple conditions in the same plate • Open system allowing real-time measurement over several days, weeks • Cost-effective • User-friendly software • Measurement in the standard culture condition	• Delay in equilibration (∼1 h) - > cannot record immediate changes • No injection system
Oroboros O2k	• Measure OCR of cells and isolated mitochondria • Records immediate changes • Unlimited by substrate injection • Cost-effective	• Can only compare two conditions simultaneously • Short-term measurements • Time-consuming • Cells are not in standard culture condition
Seahorse XF	• Screening of multiple conditions in the same plate • Measure OCR of cells and isolated mitochondria • Can measure extracellular acidification rate (ECAR) and proton efflux rate • Fully automated system	• Expensive • Preparation required the day before the experiment • Limited to four sequential injections • Cells are not in standard culture condition • Short-term measurements

In this manuscript, we present our optimized protocol for measuring mitochondrial bioenergetics using the Resipher system. Here, we describe a step-by-step optimization for monitoring the OCR using patient-derived brain tumor stem cells (BTSCs) that are maintained in 3D spheroids as cellular models. Additionally, we provide detailed instructions to adapt this protocol to other cell types. We describe detailed guidance on how to perform these analyses successfully, including determining the cell density, adjusting concentrations of the inhibitors, and analyzing the measurements. This protocol has broad applications, including, but not limited to, profiling the mitochondrial respiration of specific cell types, assessing metabolic changes upon genetic or pharmacological manipulations, evaluating the side effects or pharmacological efficacy of drugs and studying metabolic changes associated with stem cell differentiation.

### Preparation and storage of culture media

**Timing: ∼15 min**

1. Prepare 50 mL BTSC growth medium by supplementing DMEM/F12 medium with 1X B-27 supplement without vitamin A, 2 mM GlutaMAX, 100 U/mL penicillin, 100 μg/mL streptomycin, 5 mM HEPES, 2 μg/mL heparin, 20 ng/mL human EGF and 20 ng/mL human FGF as described in Table 2.

**Table 2:** Composition of BTSC growth medium

Reagent	Final concentration	Amount
DMEM/F12	N/A	47.7 mL
B-27 supplement without vitamin A (50X)	1 X	1 mL
GlutaMAX (200 mM)	2 mM	500 μL
HEPES (1 M)	5 mM	250 μL
Streptomycin - Penicillin	100 μg/mL – 100 U/mL	500 μL
Heparin (2 mg/mL)	2 μg/mL	50 μL
Human EGF (100 μg/mL)	20 ng/mL	5 μL
Human FGF (100 μg/mL)	20 ng/mL	5 μL
**Total**	N/A	∼ **50 mL**
2. Store the BTSC growth medium supplemented with growth factors (EGF and FGF) at 4°C.
3. Prior to use, bring the growth medium to 19°C–21°C for ∼1 h, or warm it up in a water bath at 37°C for 10 min.

***Note:*** Due to the low stability of growth factors, we recommend preparing media as needed rather than stocking large batches. BTSC growth media should be made fresh and used within 2 weeks.

***Alternatives:*** Multiple variants of culture media for BTSCs can be used. The NeuroCult NS-A Proliferation Kit is a growth media kit that is provided by STEMCELL Technologies (STEMCELL Technologies, #05751). Different combinations of DMEM/F12, NeuroCult NS-A basal medium (STEMCELL Technologies, #05750), or Gibco Neurobasal Medium (Thermo Fisher Scientific, #21103049) with N-2 Supplement (Thermo Fisher Scientific, #17502048) and/or Gibco B-27 Supplement (Thermo Fisher Scientific, #12587010) have also been used in the literature.^8^

### Thawing of cryopreserved BTSCs

**Timing: ∼15 min**

**Figure 1:**
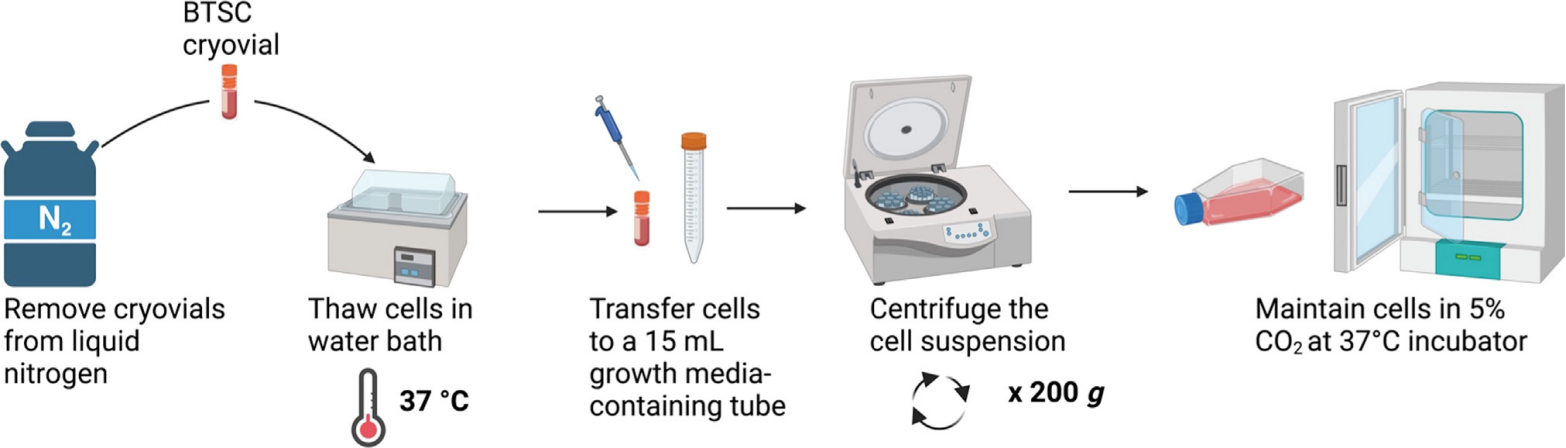
Schematic illustration of the workflow of BTSC cryovial thawing

This section describes the thawing of BTSC cryovials (Figure 1). Throughout the protocol, we used the patient-derived BTSC73 as model.

4. Thaw one cryovial of patient-derived BTSCs.
  a. Pre-warm the BTSC growth media in the 37°C water bath.
  b. Pipet 9.5 mL of pre-warmed BTSC growth media into sterile 15 mL tube.
  c. Remove the cryovial from the liquid nitrogen.
  d. Quickly transfer the cryovial to the water bath at 37°C. Do not submerge it completely to avoid water from penetrating into the cap.
  e. While holding the tip of the cryovial, gently agitate the vial for about 1 min.
  f. Remove the vial from the water bath before the complete thawing. A small ice crystal should remain.
  g. Wipe the outside of the cryovial with 70% ethanol on an absorbent paper towel, and place the cryovial under the laminar flow hood.
5. Transfer the vial content to the pre-warmed BTSC growth media.
  a. Using a P1000, aseptically transfer the semi-thawed cell suspension from the cryovial into the tube containing 9.5 mL of the pre-warmed BTSC growth media.
  b. Rinse out the cryovial once with approximately 1 mL of BTSC growth media and transfer to the 10 mL cell suspension in the tube.

***Note:*** We freeze the BTSCs in 0.5 mL BTSC growth media supplemented with 10% DMSO. If the cryovial contains a different volume of 0.5 mL, adjust the volume of the used pre-warm media in a 1:20 ratio of cell suspension to total volume to quickly dilute the DMSO to 0.5%.

6. Collect the cells by centrifugation.
  a. Centrifuge the cell suspension for 5 min at 200 × g at 20°C.
  b. Using a vacuum pump, carefully aspirate the supernatant. Do not disturb the cell pellet.
7. Seed BTSCs in BTSC growth media in low attachment flasks.
  a. Resuspend gently the cell pellet with 1 mL of BTSC growth media and transfer to a T25 flask containing 3 mL of pre-warmed BTSC growth media.
  b. Rinse the tube with approximately 1 mL of BTSC growth media and transfer the contents to the T25 flask. The T25 flask now contains the cells in 5 mL of media.

***Note:*** The size of the flasks to be used depends on the number of viable cells in the cryovials. We typically thaw cryovials containing approximately 1 million cells in a T25 flask. If you are not sure about the number of viable cells contained in the vials, proceed to cell counting as described in section “BTSC culture and maintenance”.

8. Maintain and passage the cells after 3 days.
  a. Maintain the newly thawed BTSCs in the T25 in 5% CO_2_ at 37°C in BTSC growth medium for 3 days. In the low attachment flasks, BTSCs will grow in suspension as spheres.
  b. After 3 days of thawing, passage the cells and transfer to a T75 flask as described in the next section.

### BTSC culture and maintenance

**Timing: ∼30 min**

This section describes the passaging of the newly thawed BTSCs or BTSC spheres that have been maintained in culture from previous passages. The seeding density, incubation time and growth duration may vary depending on the number of cells required for the assay and the proliferation rate of the cells.

**Figure 2:**
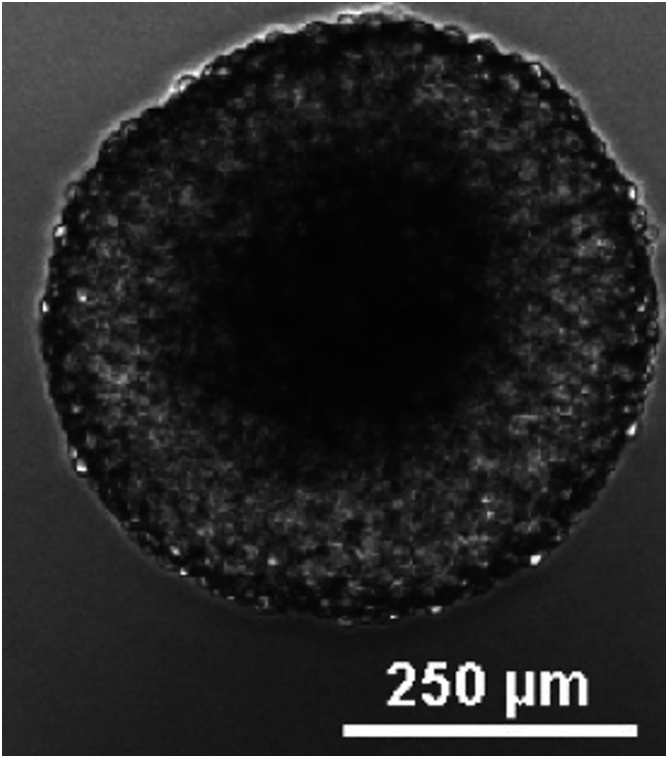
Representative image of BTSC73 spheroid that needs to be passaged is shown Scale bar: 250 μm.

***Note:*** Cells should be passaged when the neurospheres reach approximately 250–300 μm in diameter, and before the center of neurosphere darkens or the border shows signs of degradation (Figure 2).

9. Collect the cells by centrifugation.
  a. Using a 10 mL serological pipet, collect media and cells by gently mixing and washing the surface of the flask to collect all neurospheres.
  b. Transfer the media containing spheres to a 15 mL tube.
  c. Centrifuge for 5 min at 200 x g at 20°C.
10. Dissociate the cells using accutase solution.
  a. Aspirate the media.
  b. Add 200 μL of accutase solution to the cell pellet.
  c. Mix gently using a P200 micropipette.
  d. Incubate at 37°C for 10 min in the incubator. After 5 min of incubation, gently flick the tube 3–5 times to prevent the settling of the BTSC spheres at the bottom of the tube.
  e. Visually evaluate suspension, if multiple clumps seen, triturate gently 10 times using a P200 micropipette and low retention pipette tips.
  f. Add 800 μL of BTSC growth media and triturate gently using a P1000.
11. Count the number of viable cells using an exclusion dye such as trypan blue.
  a. Take 10 μL of cell suspension and mix with 10 μL of trypan blue.
  b. Transfer 10 μL of the trypan blue cell suspension mix into a Countess counter slide.
  c. Count the cells using the Countess Automatic Cell Counter.

***Alternatives:*** If an automatic cell counter is not available count the cells manually using a hemocytometer.

12. Seed cells at ∼ 0.5 × 10^6^ cells in a T75 flask in 14 mL BTSC media.
13. Maintain cells up to 7 days in culture. For BTSC73, a 7-day culture will yield around 10 × 10^6^ cells.

***Note:*** The growth rate is variable from one patient-derived BTSC line to another depending on the genetic background. Therefore, BTSC lines can be seeded at variable densities and passaged at variable frequencies based on their growth rate.

### Preparation of stock and working solutions

**Timing: ∼30 min**

Stock and working solution should be stored at −20°C. All stocks can be stored for at least six months.

14. Prepare mitochondrial modulator stock solutions according to laboratory safety rules.
  a. Prepare a stock solution of 20 mM oligomycin in DMSO (resuspend one vial of 5 mg of oligomycin in 316 μL of DMSO).
  b. Prepare a stock solution of 100 mM FCCP in DMSO (resuspend one vial of 10 mg of FCCP in 393 μL of DMSO).
  c. Prepare a stock solution of 50 mM antimycin A in DMSO (resuspend one vial of 25 mg of antimycin A in 911.25 μL of DMSO).
  d. Prepare a stock solution of 50 mM rotenone in DMSO (weight 20 mg of rotenone in 1014.1 μL of DMSO).

***Note:*** For vials with small powder quantities, it is recommended to resuspend the entire amount of powder directly. If the vials contents or the weighted amount differs from what is indicated, adjust the volume of DMSO accordingly to obtain the required concentrations.

***Note:*** Aliquot the stock solutions into convenient volumes to avoid repeated freeze-thaw cycles.

15. Prepare a range of 1000X working solutions for each mitochondrial respiration modulator.
  a. Prepare the oligomycin working solution by diluting the 20 mM oligomycin stock solution in DMSO as described in the Table 3.

**Table 3:** Preparation of oligomycin working solutions

Oligomycin solution to be prepared	Volume of oligomycin (20 mM stock solution)	Volume of DMSO
1 mM	5 μL	95 μL
2 mM	10 μL	90 μL
4 mM	20 μL	80 μL
  b. Prepare the FCCP working solution by diluting the 100 mM FCCP stock solution in DMSO as described in the Table 4.

**Table 4:** Preparation of FCCP working solutions

FCCP solution to be prepared	Volume of FCCP (100 mM stock solution)	Volume of DMSO
1 mM	1 μL	99 μL
3 mM	3 μL	97 μL
6 mM	6 μL	94 μL
  c. Prepare the rotenone-antimycin A mix working solution by diluting the 50 mM rotenone and 50 mM antimycin A stock solution in DMSO as described in the Table 5.

**Table 5:** Preparation of rotenone-antimycin A working solutions

Rotenone-antimycin A solution to be prepared	Volume of rotenone (50 mM stock solution)	Volume of antimycin A (50 mM stock solution)	Volume of DMSO
0.5 mM	1 μL	1 μL	98 μL
1 mM	2 μL	2 μL	96 μL
2 mM	4 μL	4 μL	92 μL

***Note:*** The working solutions should be aliquoted into 10 μL aliquots.

### Setting up the Resipher system

**Timing: ∼20 min**

**Figure 3:**
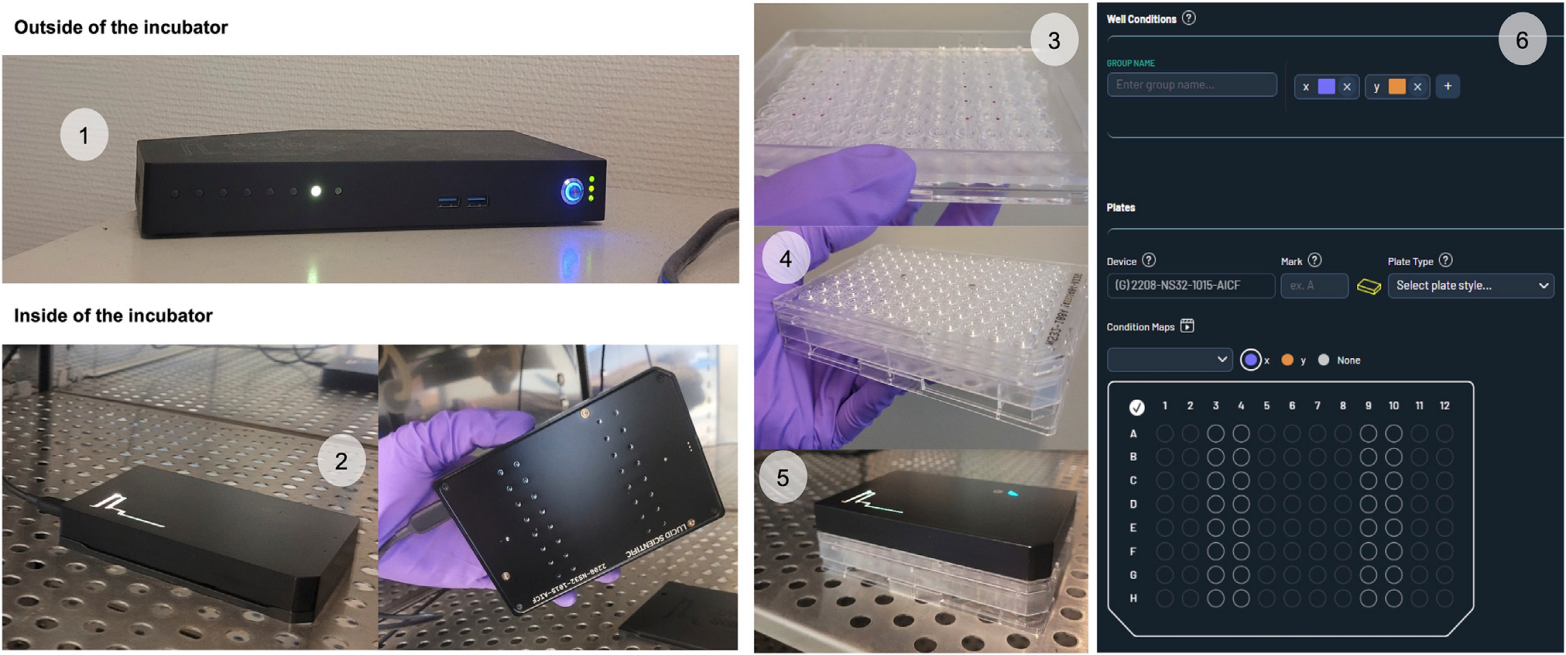
Representation of the Resipher system set-up 1: hub placed outside of the incubator, 2: Resipher placed inside the incubator, 3: lid with 32 electrodes (column 3, 4, 9 and 10), 4: sensing lid/well-plate assembly, 5: Resipher/sensing lid/well-plate assembly, 6: Lucid Lab web application layout.
17. Connect the Resipher hub to a power source and plug in an Ethernet cable to the Ethernet port on the Hub. Though the hub can function via WIFI, a wired connection through Ethernet cable is preferred.
18. Place the Resipher device into the standard humidified cell culture incubator at 37°C and 5% CO_2_.
19. Pass the USB-C cable through the opening in the back of the incubator to connect the Resipher device inside the incubator to one of the 8 USB-C ports of the Hub outside the incubator.
20. Using a computer workstation, open the Lucid lab web application: https://lab.lucidsci.com/ and create your account to be able to configure and manage your experiments and monitor data from the Resipher device in real time.

***Note:*** All the Resipher components including the Hub, the Resipher, the lid, 96 wells culture plate and the Lucid lab web application are shown in Figure 3.

16. Place the Hub onto or near the cell culture incubator (Figure 3).

## Key resources table

**Table:** 

REAGENT or RESOURCE	SOURCE	IDENTIFIER
**Chemicals, peptides, and recombinant proteins**		

4-(trifluoromethoxy)phenylhydrazone carbonyl cyanide (FCCP)	Sigma-Aldrich	#C2920
Oligomycin from *Streptomyces diastatochromogenes*	Sigma-Aldrich	#O4876
Gibco DMEM/F-12, without glutamine	Gibco	#21331020
Gibco GlutaMAX (200 mM)	Gibco	#35050061
Gibco Penicillin-streptomycin (10,000 U/mL)	Gibco	#15140122
HEPES buffered saline	Sigma-Aldrich	#51558
Heparin sodium salt	Sigma-Aldrich	#H3149
Animal-free recombinant human epidermal growth factor (hEGF)	PeproTech	#AF-100-15
Animal-free human fibroblast growth factor-basic (hFGF)	PeproTech	#AF-100-18B
Gibco Supplement B-27 (50X), without vitamin A	Gibco	#12587010
Gibco Dulbecco’s Phosphate-buffered saline (PBS), without calcium, without magnesium	Gibco	#14190094
Corning cell detachment solution Accutase	Corning	#25-058-CI
Antimycin A	Sigma-Aldrich	#A8674
Rotenone	Sigma-Aldrich	#R8875
Dimethyl sulfoxide (DMSO)	Sigma-Aldrich	#D2438
IACS-010759	Selleck Chemicals	#S8731

**Experimental models: Cell lines**		

Brain Tumor Stem Cell 73 (BTSC73)	University of Calgary^9^	N/A

**Software and algorithms**		

GraphPad Prism v.10	GraphPad	N/A
Excel	Microsoft	N/A
Lucid Lab v.1.3	Lucid Scientific	https://lab.lucidsci.com/

**Other**		

Thermo Scientific Nunc MicroWell 96-well, Nunclon delta-treated, flat-bottom Microplate	Thermo Scientific	#167008
Cell culture flask, T-75, surface: suspension, filter cap	Sarstedt	#83.3911.502
Screw cap tube, 50 mL, (LxØ): 114 × 28 mm, PP, with print	Sarstedt	#62.547.254
Screw cap tube, 15 mL, (LxØ): 120 × 17 mm, PP, with print	Sarstedt	#62.554.502
Countess II automated cell counter	Invitrogen	#AMQAX1000
Invitrogen Cell counting slides Countess	Invitrogen	#C10228
Resipher starter kit (containing Hub, Resipher and 5 sensor lids)	Lucid Scientific	N/A

## Step-by-step method details

### Determination of appropriate cell density

**Timing: ∼10 h**

This step serves to identify the appropriate cell number per well for the detection of cellular respiration for each cellular model.

**CRITICAL:** Prior to assessing changes in the OCR (induction or inhibition) in response to different experimental condition, it is crucial to determine the optimal cell density per well for each cell line. The optimal cell density may vary between different cell lines due to differences in shape, size, volume, respiration capacity and/or other biological characteristics.

**Figure 4:**
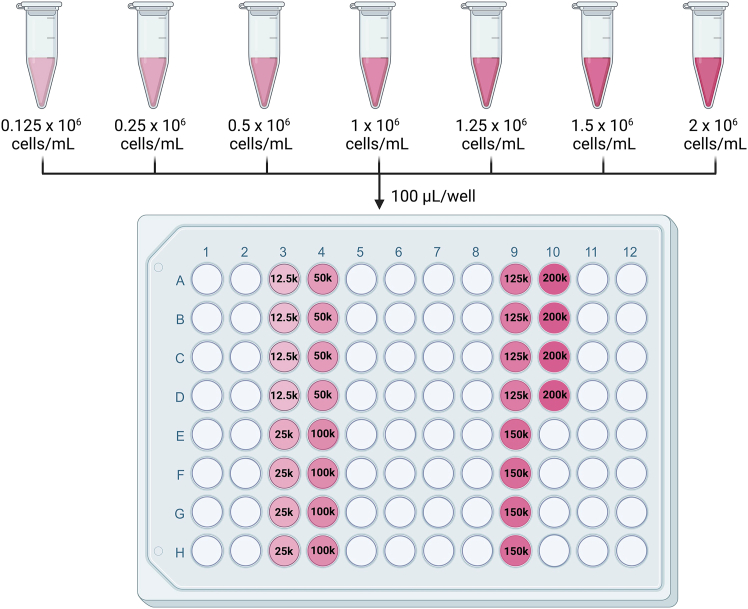
Schematic illustration of the workflow of BTSC plating to determine the appropriate cell density
    iv. Fill the unused wells with 100 μL PBS to reduce media evaporation during the assay.
    v. After plating, incubate the cells for at least 4 h at 37°C, 5% CO_2_ before starting OCR acquisition to allow the cells to settle down and form spheres.
      **CRITICAL:** This timing may need to be adjusted depending on the cell line tested. Recording can be performed after a longer incubation period; however, it is critical to ensure that the number of cells is consistent across all conditions. If the experimental conditions or treatments impact cell proliferation, recordings should be made within a time frame prior to cell division.

1. Cell counting and seeding:
  a. Collect and dissociate BTSCs.
    i. Decontaminate the biological safety cabinet with 70% ethanol and take your T75 flask out of the incubator.
      **CRITICAL:** Always work in a sterile environment and according to laboratory safety rules.
    ii. Under the biosafety cabinet, transfer the BTSCs sphere into a 15 mL tube.
    iii. Centrifuge BTSCs at 200 x g for 5 min at 20°C.
    iv. Discard the medium and add 200 μL of accutase to the pellet. Using a P200 pipette gently triturate the cells.
    v. Incubate at 37°C, 5% CO_2_ for 10 min.
    vi. Following the incubation period, add 800 μL of BTSC growth media.
    vii. Dissociate the cells by pipetting up and down several times.
  b. Count the number of viable cells using an exclusion dye such as trypan blue.
    i. Take 10 μL of cell suspension and mix with 10 μL of trypan blue.
    ii. Transfer 10 μL of the trypan blue cell suspension mix into a Countess counter slide.
    iii. Count the cells using the Countess Automatic Cell Counter.
  c. Seed the cells at varying densities, as indicated in Table 6, into a 96-well cell culture plate that is compatible with the Resipher sensing lids.
    ***Note:*** We recommend to run at least 4 replicates of each condition.
    i. Transfer the volume containing the required number of cells for 5 replicates (4 replicates and one extra for pipetting error) per condition into an Eppendorf tube.
    ii. Centrifuge each tube at 200 x g for 5 min at 20°C, discard supernatant and resuspend the cell pellet in 500 μL of fresh BTSC growth media.
    iii. Homogenize cells and seed 100 μL of cell suspension in quadruplicate in a 96-well Nunc plate (Figure 4). The total amount of cells needed for this experiment is 3.32 million cells.
      ***Note:*** The sensing lids of Resipher are designed to fit specific plate styles and should only be used with the corresponding compatible plates. Using non-compatible plates may damage the sensing lids. For a complete list of compatible plates, refer to the Lucid Scientific guide for the catalog numbers of compatible 96-well plates. Throughout this protocol, we used the Nunc, clear, flat bottom 96-well plates.
      **CRITICAL:** When using the 32-channel Resipher OCR reader, cells should be plated in columns 3, 4, 9 and 10 of the 96-well plate. The 32-channel Resipher sensing lid is equipped with probes only for these specific columns only.

**Table 6:** Cell dilution preparation

Conditions (cells seeded/well)	12.5k	25k	50k	100k	125k	150k	200k
Number of cells needed for 5 replicates per condition	62.5k	125k	250k	500k	625k	750k	1.10^6^
2. At least 1 h before running the Resipher assay, pre-warm the Resipher sensing lid.
  a. Under a biological safety cabinet, fill a 96-well plate with 100 μL sterile PBS per well. At least columns 3, 4, 9 and 10 should be filled with PBS.
  b. Open the Resipher sensing lid package, and gently place the sensing lid onto the PBS containing plate. Make sure that the sensor probes are immersed in PBS.
    ***Note:*** When placing the sensing lid on the plate, gently lower the sensing probes into the wells, avoiding contact with the well borders. Direct contact of the probe with the well can damage the oxygen sensing probes and lead to failed experiments.
  c. Incubate the sensor lid on the PBS plate in the cell culture incubator at 37°C and 5% CO_2_ for at least 1 h.
    **CRITICAL:** This step is essential as it pre-warms the sensing lids. The oxygen-sensing probes are temperature and humidity dependent, and pre-warming brings them to the incubator temperature (∼37°C). This ensures rapid stabilization of the OCR recordings after initiation of acquisition.
3. Run Resipher acquisition.
  a. Log in to your account in Lucid lab web app: https://lab.lucidsci.com/ and click the “+” button in the upper right to create a new experiment.
  b. Enter the title for your experiment.
    ***Optional:*** You may add experimental details in the “Summary” section.
  c. Define the experimental groups in the “Well conditions” section.
    i. Type in the “Group name”. This should correspond to the varying experimental conditions, such as the cell density or treatment.
    ii. Type in the “Values“. The values represent the specifics of your experimental condition such as the number of cells seeded per well, or the concentrations of drug.
      ***Note:*** For example, for this experiment, in the box “Group name” write “Cell density”, and in the box “Values” write the number of cells seeded per well, as outlined in step 1.c , “12.5k”, “25k”, “50k”, “100k”, “125k”, “150k” and “200k”.
      ***Note:*** New values can be added by clicking the “+” button in the “Well conditions” section.
      ***Optional:*** Default colors of the values can be modified.
  d. Scroll down to “Add plate” button to configure the plate.
    ***Note:*** If you are using only one Resipher device on your account, the “device” section will be filled automatically. If multiple devices are connected on the same account select the correct device from the drop down menu.
    i. Type in the “Mark”.
      ***Note:*** The “Mark” indicator is optional when using a single device. However, the “Mark” indicator can be used when multiple devices are used at the same time to identify plates and devices. The “Mark” corresponds to the alphabet letter on the back of each Resipher device.
    ii. Select the “Plate Type” you are using from the drop-down menu.
    iii. On the “Condition Maps” select experimental groups and attribute groups and values to each well.
      ***Note:*** Labeling each well with the appropriate group information and color is essential to ensure the correct grouping of the wells, when following the progression of the experiment in real time or during data analysis.
    iv. Save the experiment by clicking the “Save” button for later use.
  e. Place the sensing lid on the cell culture plates to initiate the experiment.
    i. Retrieve the pre-seeded 96-well plate from the incubator and place under the biosafety cabinet. The cells should have been incubated for at least 4 h (from step 1.c.v).
    ii. Retrieve the pre-warmed sensing lid on the PBS plate from the incubator and place it under the biosafety cabinet.
      ***Note:*** The sensing lid should have been pre-warmed for at least 1 h (from step 2.c).
    iii. Under the biosafety cabinet, gently place the sensing lid onto the cell culture plate.
    iv. Transfer the sensing lid/well-plate assembly to the incubator and attach the Resipher device to the top of the sensing lid.
      ***Note:*** The magnets embedded in both the device and the lid ensure a proper and tight fit. Once the device and the sensing lid are correctly attached, the indicator LEDs on both the Resipher and the Hub will flash green. The LEDs will turn solid green once the Resipher actuation begins and stabilizes.
    v. On the Lucid Scientific web app: https://lab.lucidsci.com/.
    vi. Go to the saved experiment from step 3.d.v and click on “Start now” to begin the Resipher acquisition.
      ***Note:*** The experiment status will change to “In Progress”, and the data will be securely stored on both the Hub and in the cloud.
      ***Optional:*** Creating and saving a new experiment before attaching the Resipher to the cell culture plate is not required. The experiment can be created and launched immediately after attaching the Resipher to the plate.
    vii. To monitor the data generated by the experiment in real-time, log into the Lucid lab web app and click on the experiment in progress. The OCR measurements will be displayed in real-time.
    viii. A first inspection of the results can be performed at this point. If high standard deviations within one condition are observed, switching from the “average” view to the “raw” view can help identify potential outliers (Figure 5). Outliers may result from issues such as inaccurate plating or error in the sensing probes.

**Figure 5:**
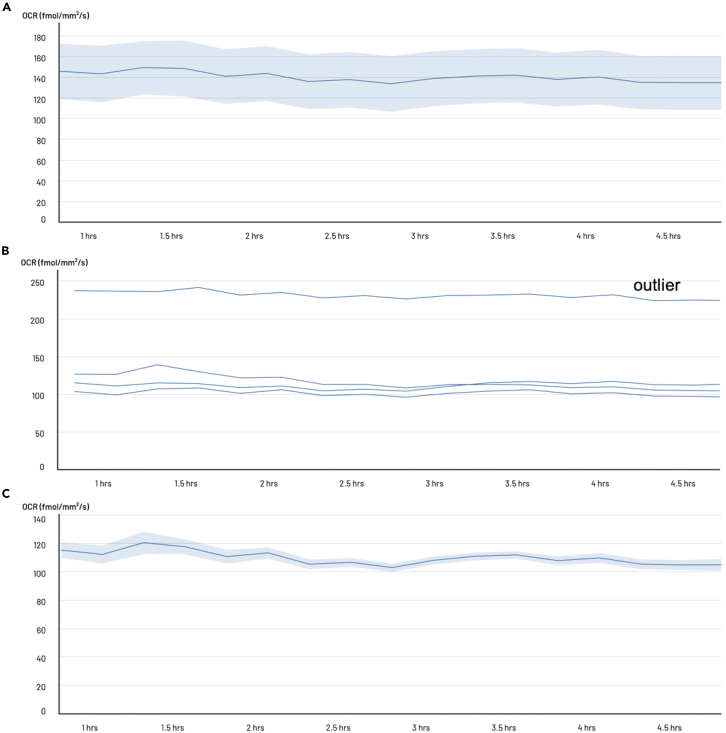
Identification and removal of an outlier (A) Large variability is observed within a single condition, indicating the presence of an outlier. (B) The visualization mode is switched from “average” to “raw” view to display individual data points, which aids in more clearly identifying the outlier. (C) The identified outlier is removed from the dataset to improve the overall analysis.
    ix. Wait for the OCR curves to be stable. Stability is often achieved within the first 1–2 h of acquisition (Figure 6).

**Figure 6:**
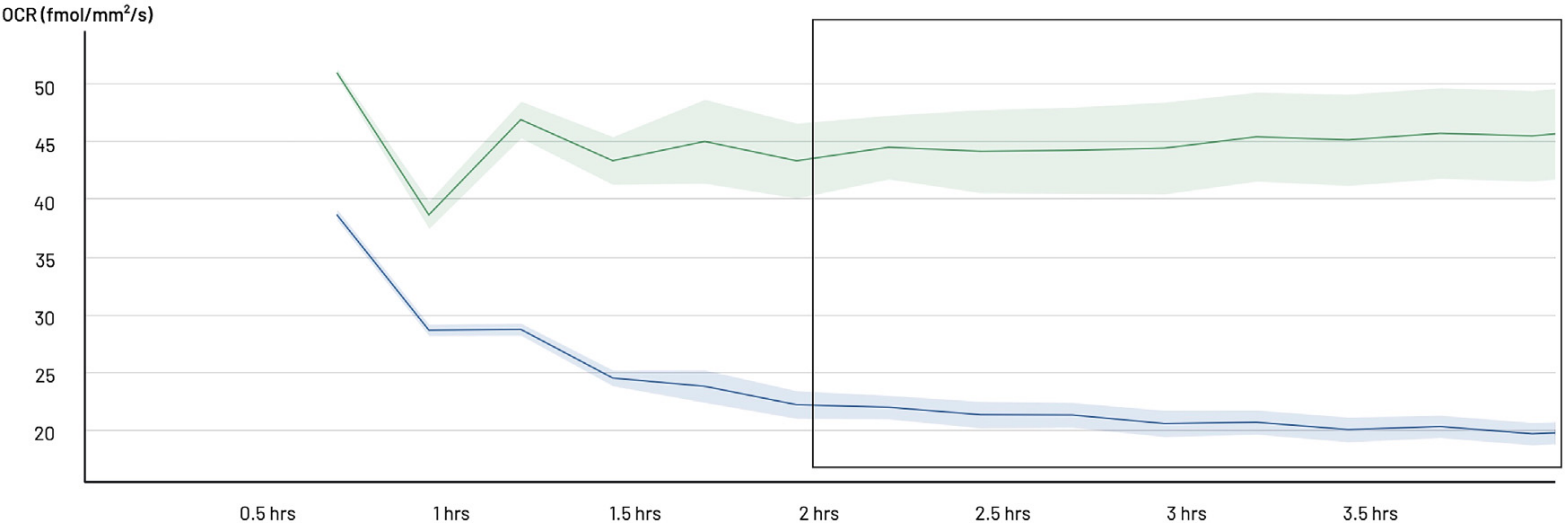
Example of OCR acquisition The signal stabilizes after 2 h, at which point reliable analysis can be performed.
    x. At the end of the assay, click on the “End experiment” button to end the experiment.
      ***Optional:*** Although the sensing lids are provided by Lucid Scientific as single-use consumables, they can be reused if properly cleaned and maintained. At the end of the assay, if reuse is intended, retrieve the sensing lid and place it in a 96-well plate filled with 70% ethanol for 30 min to clean the sensing probes. After cleaning, store the sensing lid in an empty 96-well plate, in the dark protected from light exposure.
4. Data analysis:
  a. Retrieve data generated by the experiment.
    i. Open the Lucid lab website: https://lab.lucidsci.com/.
    ii. Choose your experiment and export the data by clicking the “Export data”. A window to select the different export parameters will pop out.
    iii. Select the desired parameters. We usually keep the parameters as selected by default, which are as follows: Data Format, Wide; File Format, CSV; Compression, None; Measure, Flux; Resample Data, intervals 15 min, method resample (average).
  b. Open the CSV file by Excel and proceed to data analysis.
    i. Collect 4–5 consecutive readings after stabilization of the signal. These five consecutive readings cover a 1-h period, if data are exported with “Resample Data” setting at 15-min intervals.
    ii. Calculate the mean of the 5 consecutive readings for each well, and then calculate the mean of the 4 replicates (wells) for each condition.
    iii. Plot data using Excel or Prism.

**Figure 7:**
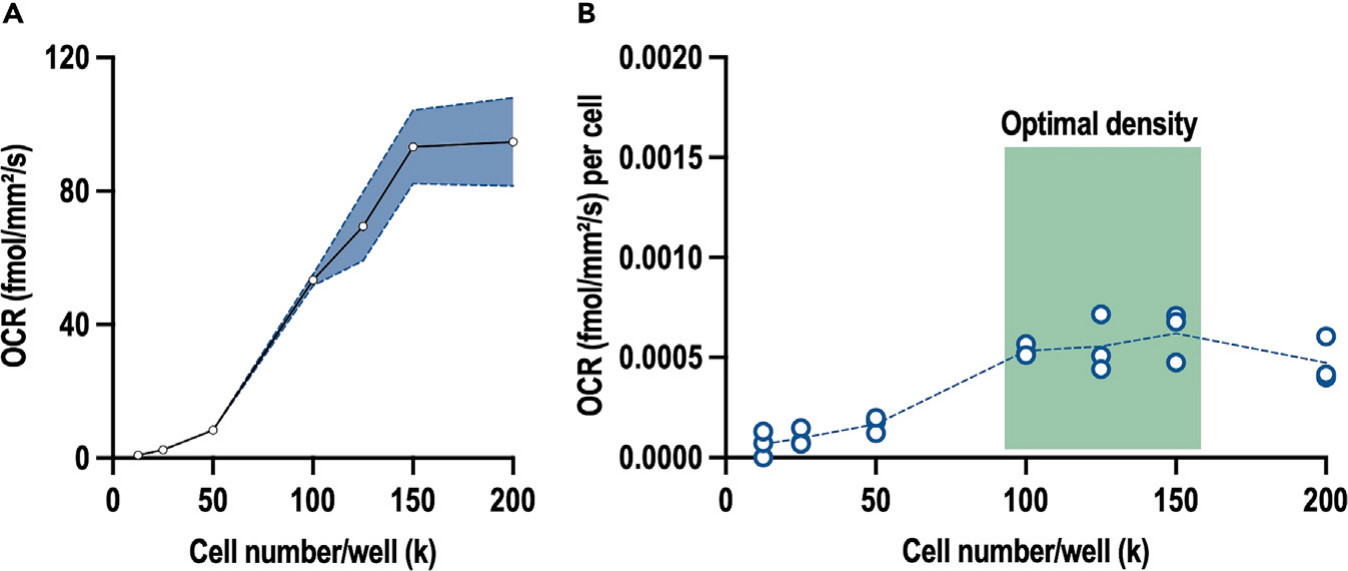
Identification of the appropriate cell density (A and B) BTSC73 cells were cultured at varying densities per well and subjected to OCR analysis to determine the optimal density for subsequent experiments. The OCR per well at different cell density is represented to assess the linearity of OCR in relation to cell density (A). The OCR per cell, calculated by normalizing the OCR data to the number of cells, is represented (B). Data are presented as the means ± SEM, *n* = 3 independent biological experiments.

***Note:*** The aim of this experiment was to determine the appropriate cell density for the BTSC73 cell line. The optimal cell density was found to be between 50k and 150k cells, and 125k per well were selected for downstream analysis (Figure 7). Refer to the expected outcome for interpretation.

### Determination of the optimal concentration of mitochondrial respiration modulators

**Timing: ∼10 h**

This step involves the careful titration of oligomycin, FCCP and rotenone-antimycin A to determine the optimal concentrations for each mitochondrial respiration modulator in each cell line. The aim is to identify the lowest concentration of each compound that produces the maximum effect without causing cell damage.

5. Cell counting and plate seeding:
  a. Dissociate and count the BTSCs as described in step 1a-b.
  b. Seed BTSCs at the selected density. The determination of the optimal cell number per well is described in the previous section. The selected density for BTSC73 is 125k cells per well.
  c. Calculate the required number of cells needed and the corresponding volume of the cell suspension to be plated for this step.
    ***Note:*** For each concentration of mitochondrial respiration modulator (FCCP, oligomycin and rotenone- antimycin A), at least 4 replicates (4 wells) should be tested. As shown in the table below, 4 concentrations were tested for each mitochondrial modulator. The total number of cells required for this type of experiment is approximately 7 million.
    ***Note:*** If more than 32 wells (i.e., more than 1 plate) are needed to cover all the conditions, the samples can be run on multiple plates. However, a control should be repeated on each plate.
    i. Transfer the volume of the cell suspension containing the required number of cells into a new tube and centrifuge the BTSCs at 200 x g for 5 min et 20°C.
    ii. Discard the supernatant and resuspend the cells in the required volume to obtain 125k cell/75 μL in growth media. Ensure a homogeneous cell suspension by gently triturate the cells using a pipette.
    iii. Plate 75 μL in each well of 96-well Nunc plates.
    iv. Incubate the cells at least for 4 h at 37°C, 5% CO_2_ before adding the mitochondrial modulators.
6. Prepare mitochondrial modulators at 4X concentration in BTSC growth media as follows:
  a. Thaw and vortex the working solution (1000X) of the mitochondrial modulators:
    i. FCCP: 1 mM, 3 mM and 6 mM.
    ii. Oligomycin: 1 mM, 2 mM and 4 mM.
    iii. Rotenone-antimycin A: 0.5 mM, 1 mM and 2 mM.
  b. Prepare 125 μL at 4X mitochondrial respiration modulators by adding 0.5 μL of each 1000X working solution to 124.5 μL growth media (Table 7).

**Table 7:** Preparation of mitochondrial respiration modulator solutions

Compound	Initial concentration (1000X working solution)	Concentration of the intermediate (4X working solution)	Final concentration in the well	Final percentage of DMSO
Oligomycin	1 mM	4 μM	1 μM	0.1%
	2 mM	8 μM	2 μM	0.1%
	4 mM	12 μM	4 μM	0.1%
FCCP	1 mM	4 μM	1 μM	0.1%
	3 mM	12 μM	3 μM	0.1%
	6 mM	24 μM	6 μM	0.1%
Rotenone-antimycin A mix	0.5 mM	2 μM	0.5 μM	0.1%
	1 mM	4 μM	1 μM	0.1%
	2 mM	8 μM	2 μM	0.1%
  c. Vortex each 4X mix and treat the cells from step 5.c.iv with 25 μL per well (Figure 8). The final volume per well will be 100 μL and each compound at a final concentration of 1X.

**Figure 8:**
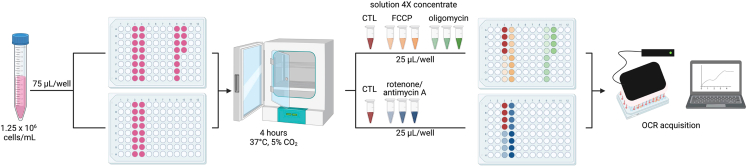
Schematic illustration of the workflow of BTSC treatment with the mitochondrial respiration modulators

***Note:*** By diluting all the compounds as described above, the final percentage of DMSO will be the same in all conditions. If different scenario of treatment is to be used, make sure to adjust the final amount of DMSO between conditions.

7. Run the Resipher acquisition as described above (step 3) with the following modifications:
  a. For plate 1, enter three “Group name”: control, FCCP and oligomycin. For plate 2, enter two “Group name”: control and rotenone-antimycin A.
  b. Type in the “Values”. For plate 1, the value for control group is 0; the values for FCCP group are: 1 μM, 3 μM, 6 μM; and the values for oligomycin group are: 1 μM, 2 μM and 4 μM. For plate 2, the value for control group is 0 and the values for rotenone-antimycin A group are: 0.5 μM, 1 μM, 2 μM.
  c. Place the pre-warmed sensing lid on the treated cells as described in step 3.e.
  d. Initiate OCR recording by clicking on “Start Experiment” on the Lucid lab web app: https://lab.lucidsci.com/.
8. Retrieve the data by logging to the Lucid lab web app and analyze data as described in step 4.

**Figure 9:**
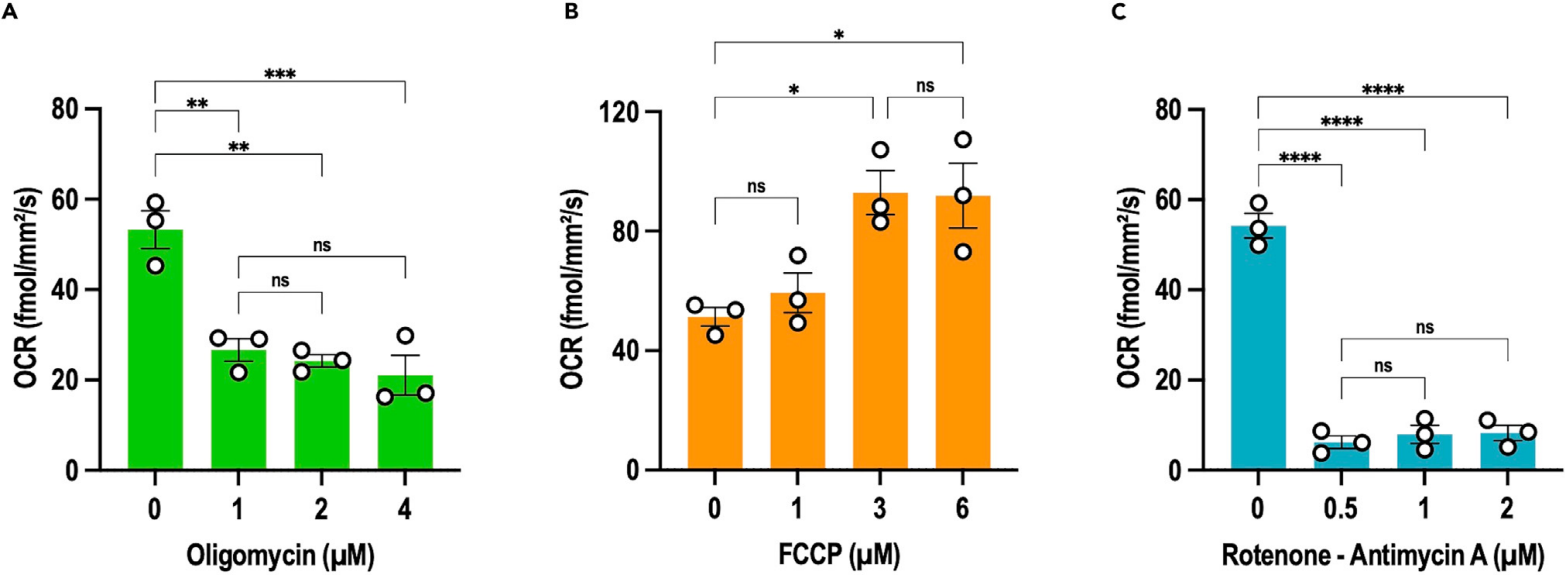
Titration of mitochondrial respiration modulator concentrations (A–C) BTSC73 cells were treated with different concentrations of oligomycin (A), FCCP (B), or rotenone-antimycin A (C) and subjected to OCR analysis using the real-time Resipher system. Data are presented as the means ± SEM, *n* = 3 independent biological experiments. One-way ANOVA followed by Tukey’s test, ∗*p* < 0.05, ∗∗*p* < 0.01, ∗∗∗*p* < 0.001, ∗∗∗∗*p* < 0.0001.

***Note:*** The aim of this step is to determine the optimal concentration of each mitochondrial respiration modulator for the cell line of interest. For BTSC73 cells, the optimal concentrations are 1 μM of oligomycin, 3 μM of FCCP and 0.5 μM of rotenone-antimycin A (Figure 9). Refer to the expected outcome for interpretation.

### Run the experiment

**Timing: ∼10 h**

After establishing the optimal cell density and the optimal concentration of each mitochondrial respiration modulator in each cell line, proceed to run the assay test. This protocol has broad applications such as profiling the mitochondrial respiration of different cell types or assessing metabolic changes in response to genetic or pharmacological interventions. Here, as an example, we aimed to assess the changes in the mitochondrial bioenergetics of BTSCs in response to treatment with the pharmacological OXPHOS inhibitor IACS-010759. Another example of such an application of pharmacological manipulation, can be found in our recent study.^1^

9. Cell counting and plate seeding:
  a. Dissociate and count BTSCs as described in section “BTSC culture and maintenance”.
  b. Plate 125k cells in 75 μL per wells in columns 3, 4, 9 and 10 of 96-well Nunc plates as described in step 5c.
  c. Incubate the cells for at least 4 h at 37°C, 5% CO_2_ before adding 20 nM IACS-010759 and/or the mitochondrial modulators at the appropriate concentrations.
  d. Pre-warm the sensing lid as described in step 2 for at least 1 h.
10. Treatment with IACS-010759 and the mitochondrial respiration modulators:
  a. Prepare 8 Eppendorf tubes, each containing 125 μL of BTSCs growth media.
  b. Thaw and vortex the 1000X IACS-010759 working solution (20 μM in DMSO). Add 0.5 μL of the 1000X IACS-010759 to 4 of the tubes to obtain 4X intermediate IACS-010759 solution. Label the tubes as IACS-010759.
  c. Add 0.5 μL of DMSO to the remaining 4 tubes and label them as control.
  d. Thaw and vortex the following 1000X working solutions of the mitochondrial respiration modulators:
    i. Oligomycin: 1 mM.
    ii. FCCP: 3 mM.
    iii. Rotenone-antimycin A: 0.5 mM.
  e. Take one control tube and one IACS-010759 tube, and add 0.5 μL of the 1 mM oligomycin solution to each. Label them as control oligomycin and IACS-010759 oligomycin, respectively.
  f. Take another control and IACS-010759 tube, and add 0.5 μL of the 3 mM FCCP solution to each. Label them as control FCCP and IACS-010759 FCCP, respectively.
  g. Take the third control tube and the third IACS-010759 tube and add 0.5 μL of the 0.5 mM rotenone A-antimycin A solution to each. Label them as control rotenone-antimycin A and IACS-010759 rotenone-antimycin A, respectively.
  h. In the remaining control tube and IACS-010759 tube, add 0.5 μL DMSO. Keep their initial label as control and IACS-010759.
  i. Vortex each preparation and treat the cells (from step 8) in quadruplicate for all the conditions.

***Note:*** By following this treatment scenario, the final percentage of DMSO will be the same in all the wells. If a different treatment scenario is used, be sure to adjust the DMSO concentration accordingly between conditions.

**Figure 10:**
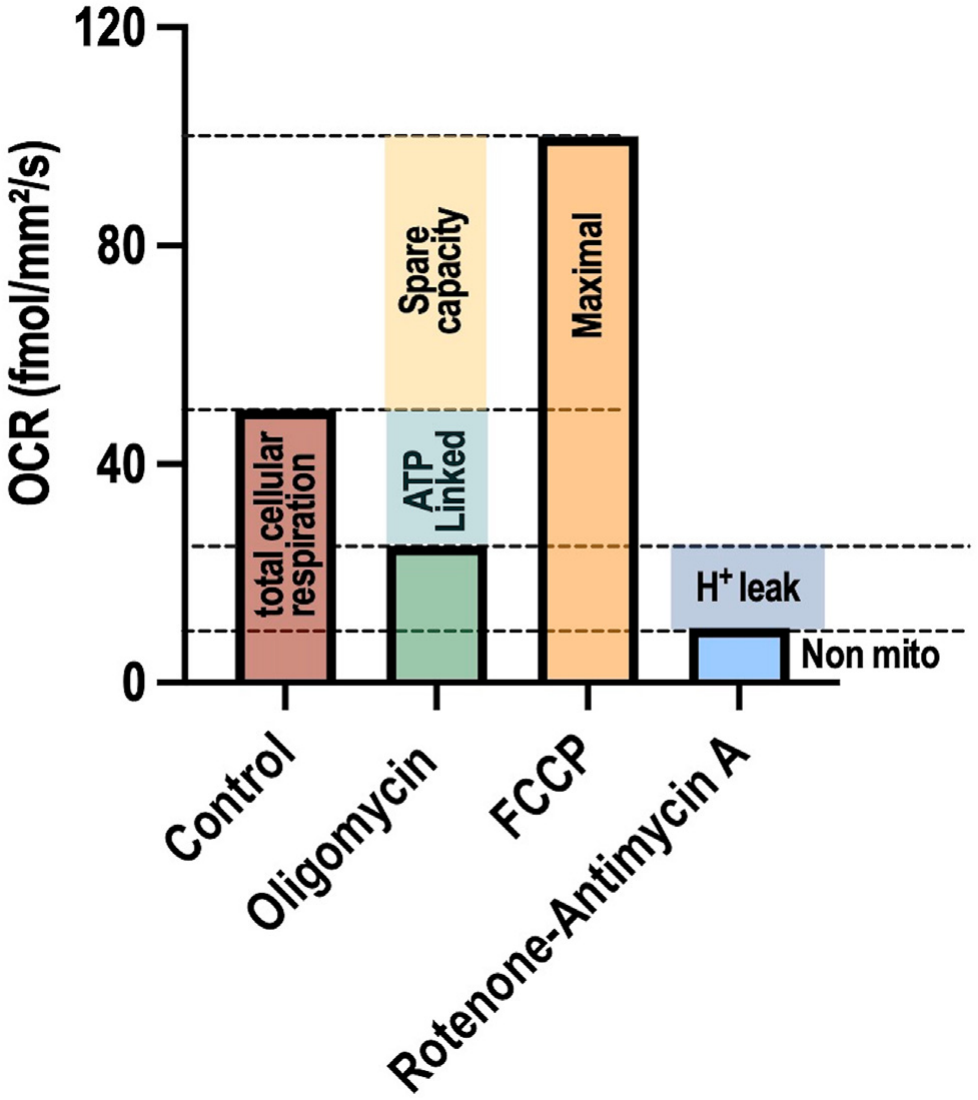
Schematic illustration showing the calculation of the respiratory parameters

11. Run the Resipher acquisition as described in step 3 with the following modifications:
  a. On the Lucid lab web app, create a new experiment.
  b. Assign the “Group name” and “Values”.
  c. Save the experiment.
  d. Gently place the pre-warmed sensing lid to the cell culture plate.
  e. Transfer the sensing lid/96-well plate assembly to the incubator and attach the Resipher device to the top of the sensing lid.
  f. Initiate the saved experiment by clicking on “Start experiment”.
  g. Monitor the OCR by logging in to the Lucid lab web.
  h. At the end of the assay, end the experiment by clicking on “End experiment”.
12. Data analysis:
  a. Retrieve data generated by the experiment in a CSV file and proceed to data analysis as in step 4.
  b. From these data, the following parameters can be determined for each experimental condition (Figure 10):
    i. Total cellular respiration: the OCR in the absence of any mitochondrial respiration modulators. Basal mitochondrial respiration can be precisely determined through the difference between the total cellular OCR and the OCR of cells treated with rotenone-antimycin A.
    ii. Non-mitochondrial respiration: the OCR in cells treated with rotenone-antimycin A. Rotenone is a complex I inhibitor, and antimycin A, a complex III inhibitor. The combination of these two compounds shuts down mitochondrial respiration, leaving only the oxygen consumption driven by a subset of cellular enzymes that continue to utilize oxygen despite the inhibition of mitochondrial complexes.
    iii. ATP-linked respiration: the difference between total oxygen consumption and OCR in oligomycin-treated cells. Oligomycin is an inhibitor of ATP synthase and blocks ATP production. This measure represents the portion of mitochondrial respiration that is used to drive ATP production.
    iv. Proton leak: the difference in OCR between oligomycin-treated cells and rotenone-antimycin A-treated cells. This reflects respiration associated with the proton leaking through the inner membrane, short circuiting the classic proton cycle.
    v. Maximal respiration: the OCR at its peak in FCCP-treated cells. FCCP is an uncoupling agent that mimics a physiological “energy demand” by stimulating the respiratory chain to operate at maximum capacity, leading to rapid oxidation of substrates to meet this increased demand. This measurement reflects the maximum rate of oxygen consumption that the cell can achieve. Maximal mitochondrial respiration can be determined by subtracting to this value the OCR of rotenone-antimycin A treated cells (non-mitochondrial respiration).
    vi. Spare respiratory capacity (SRC): the difference between the maximal and the total oxygen consumption, indicating the reserve capacity of mitochondria to increase respiration when needed.

## Expected outcomes

This protocol describes real-time measurement of OCR using the Resipher system in BTSCs cultured in 3D spheroids. The first crucial step is determining the optimal cell density to plate in a 96-well plate for the Resipher assay. The optimal density should provide OCR readings sensitive enough to detect both inhibition and induction of OCR.

As shown in Figure 7, a linear relationship between OCR and cell number is observed, indicating that OCR is tightly correlated with the number of cells per well. However, the optimal density range for measuring OCR, should be considered in the range where maximum oxygen consumption per cell is achieved. In Figure 7B, at cell densities below 100k cells per well, the OCR per cell does not reach its maximum value. The highest OCR per cell is observed at a cell density of 100k-150k per well. Between 100k and 150k cells per well, no significant difference in oxygen consumption per cell is observed, suggesting that this range is optimal for measuring OCR. At densities higher than 150k cells per well, specifically at 200k, a decrease in OCR per cell is observed, indicating that the system becomes saturated and OCR measurements may no longer be accurate.

**Figure 11:**
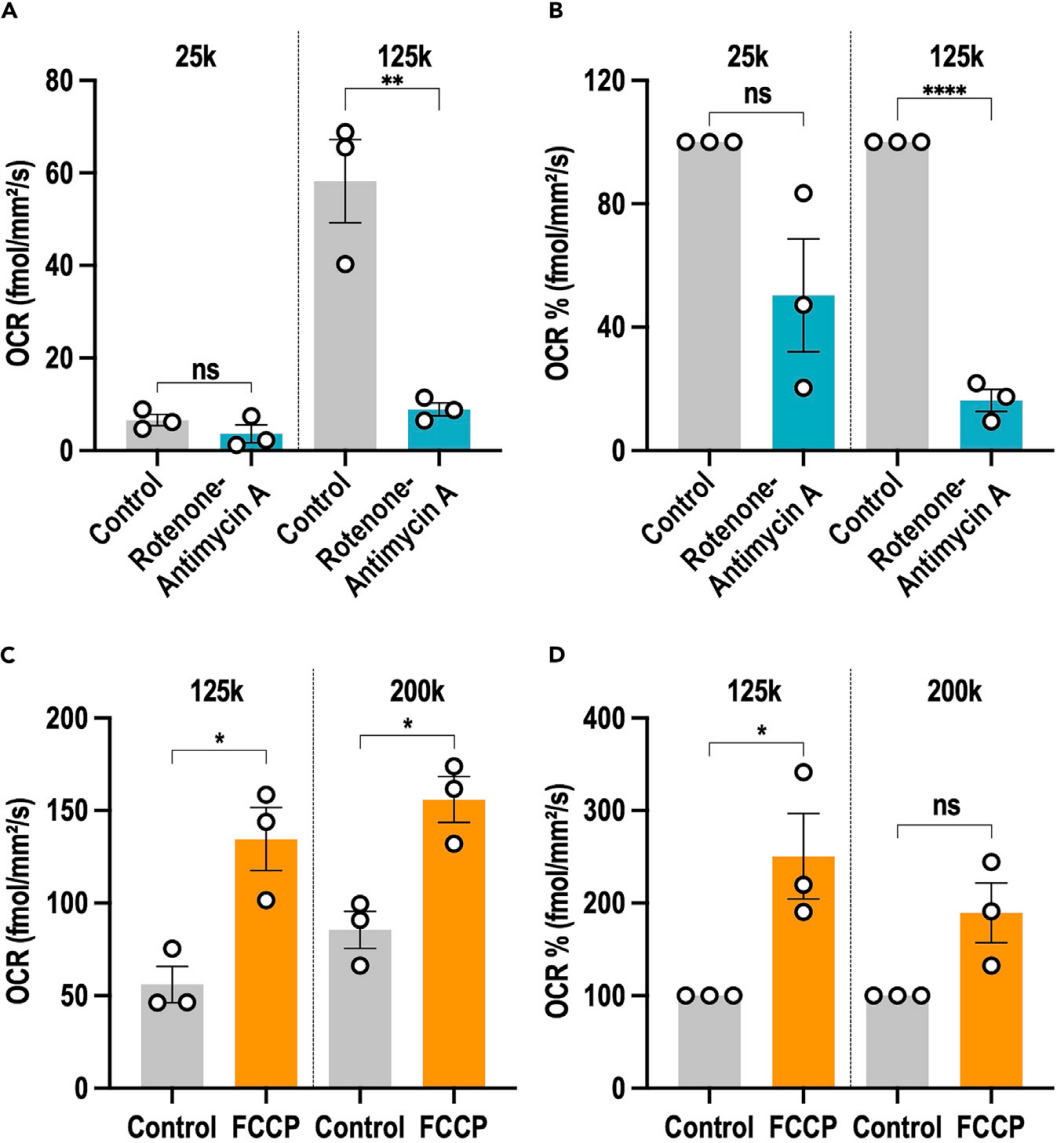
Correlation of cell density and response to mitochondrial respiration modulators (A and B) BTSC73 cells, at a density of 25kor 125k cells per well, were treated with rotenone-antimycin A and subjected to OCR analysis. Raw data are presented in (A). Data normalized to the control of each cell density are presented in (B). (C and D) BTSC73 cells, at a density of 125k or 200k cells per well, were treated with FCCP and subjected to OCR analysis. Raw data are presented in (C). Data normalized to the control of each cell density are presented in (D). Data are presented as the means ± SEM, *n* = 3 independent biological experiments. Unpaired two-tailed t test, ∗*p* < 0.05, ∗∗*p* < 0.01, ∗∗∗∗*p* < 0.0001.

Therefore, the optimal cell density per well for BTSC73 cells should fall within the 100k–150k range to ensure reliable and accurate OCR data. At lower cell densities, OCR measurements are reduced, making comparisons between conditions potentially unreliable, particularly when assessing OCR inhibition in response to metabolic interventions. For instance, at a cell density of 25k per well, rotenone-antimycin A treatment induced only a slight reduction in OCR. In contrast, at 125k cells per well, rotenone-antimycin A caused an 80% reduction in OCR (Figures 11A and 11B).

Conversely, a high BTSC73 cell density, above 150k per well, results in a saturated OCR signal, where a plateau in OCR readings is observed, limiting the ability to detect further increases in response to metabolic interventions. For example, following FCCP treatment, OCR increased 2.5-fold at 125k cells per well, but only 1.8-fold at 200k cells per well (Figures 11C and 11D). Thus, determining the optimal cell density for each cell line is crucial for ensuring accurate and reliable measurement of OCR changes.

The second essential step in this protocol is determining the optimal concentration of each mitochondrial modulator. The optimal concentration is the lowest amount of each modulator that induces the maximal effect. For this cell line, the maximal effects are achieved with the following concentrations: 1 μM oligomycin, 3 μM FCCP, and 0.5 μM rotenone-antimycin A (Figure 9).

**Figure 12:**
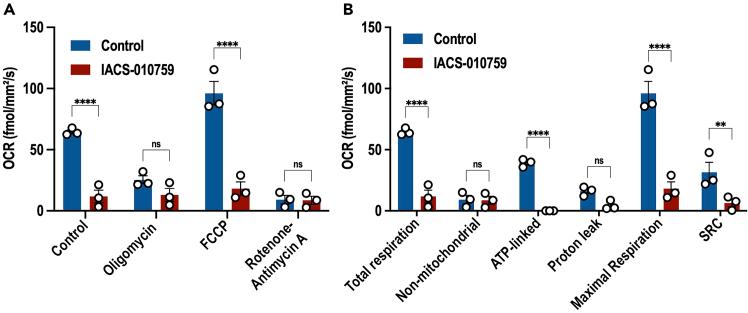
Effect of IACS-010759 on the different respiration parameters (A and B) BTSC73 cells, at a density of 125k, were treated with IACS-010759 in the absence or presence of oligomycin, FCCP, or rotenone-antimycin A (A) and subjected to Resipher analysis to determine the respiration parameters (B). Data are presented as the means ± SEM, *n* = 3 independent biological experiments. Two-way ANOVA followed by Sidak’s test, ∗∗*p* < 0.01, ∗∗∗∗*p* < 0.0001.

Finally, we present an example of OCR measurement in BTSCs following treatment with IACS-010759, a potent and selective small-molecule complex I inhibitor.^10^ As expected, our results showed a significant reduction in total cellular respiration in BTSCs after treatment with IACS-010759 (Figure 12). As described earlier, the raw data were used to calculate key bioenergetic parameters of mitochondrial function. Compared to vehicle control, treatment of BTSC73 cells with 20 nM IACS-010759 led to a significant decrease in total cellular respiration, maximal respiration, spare respiratory capacity, and ATP-linked respiration (Figure 12).

## Limitations

One limitation of the Resipher system is that the OCR signal stabilization may take from several minutes to 2 h, meaning that immediate changes in mitochondrial respiration following experimental manipulations may not be detectable. Another consideration is that a blue light flashes in the wells during OCR recordings. While this did not affect our experiments, it should be taken into account when working with photosensitive cells. Additionally, in this protocol, mitochondrial modulators are not sequentially injected into the same well; each modulator of mitochondrial respiration is tested in separate wells. While adding different modulators sequentially to the same well could be feasible, it is not automated. This process would require manual intervention, necessitating pausing the experiment, removing the plate, adding the new modulator, and waiting again for reading stabilization.

## Troubleshooting

### Problem 1

The measured OCR values are very low (-⋈ - 5), following step 3 Resipher acquisition.

### Potential solution

• Examine cells under the microscope to ensure proper seeding before step 3.e.iii.
• Use an optimal number of cells, as low cell density may result in respiration levels below the sensitivity of the Resipher system. The optimal cell density should be determined for each cell line (as explained in section “Determination of cell density”).
• After step 3.e.x, when the acquisition is complete, remove the lid and collect the cells from the plate to evaluate cell viability, as treatments or pharmacological manipulations may lead to cell death.

### Problem 2

A delay in the stabilization of OCR readings is observed following step 3 Resipher acquisition.

### Potential solution

• The sensing probes are dependent on temperature and humidity. Ensure that the sensing lid is pre-warmed in a PBS plate at 37°C with 5% CO_2_ prior to initiating the experiment (as described in section 2). Additionally, pre-warm the media containing the mitochondrial modulators before applying it to the cells after the step 6b.
• If issue persists, replace the sensing lids as the sensing probes may be damaged. Sensing lids are available at https://lucidsci.com/products.

### Problem 3

There is no data recorded for one of the wells following step 3 Resipher acquisition.

### Potential solution

• Check if the corresponding probe is damaged. The sensing probes are fragile, so handle them with care. Gently place the sensing lids over the culture plates and avoid contact with solid surfaces during storage.
• After step 3.e.x, when the acquisition is complete, examine the corresponding well under the microscope, as cell clumping may occur, causing contact with the sensing probes.
• The Resipher sensing probes move up and down during the measurements in a range of 500 μm. The sensing probes moves as close as 500 μm of the bottom of the well. Before step 3.e.iii., ensure that the spheres or cells are fully settled before placing the sensing lid on the culture plate to prevent contact with the sensing probes.

### Problem 4

Significant variation is observed in the OCR values between replicates in step 3.e.viii.

### Potential solution

• After step 3.e.x, when the acquisition is complete, check for any anomalies in cell seeding.
• Fill the unused wells with PBS to prevent evaporation as explained in step 1.c.iv.
• Avoid opening the incubator during the experiment, as the sensing probes are temperature-dependent. Fluctuations in incubator temperature may lead to measurements that do not accurately reflect the true OCR.

## Resource availability

### Lead contact

Further information and requests for resources and reagents should be directed to the lead contact, Dr. Ahmad Sharanek (ahmad.charanek@u-bordeaux.fr).

### Technical contact

Questions regarding the technical specifics of performing the protocol should be directed to the technical contacts Dr. Audrey Burban (audrey.burban@cnrs.fr) Dr. Ahmad Sharanek (ahmad.charanek@u-bordeaux.fr).

### Materials availability

This study did not generate new unique reagents.

### Data and code availability

This study did not generate any unique datasets or code.

## Acknowledgments

This work was supported by grants from Fondation de France No. 00130891 to A. Burban and No. 00130896 to A.S., Cancéropôle GSO No. 2023-E5 to A.S., and Association pour la Recherche sur les Tumeurs Cérébrales No. 283008 to A. Burban. A.S. and A. Burban were supported by a fellowship from the Fondation de France. A.S. is also a recipient of the Marie Skłodowska-Curie Actions fellowship. C.T. is a recipient of the NewMoon PhD fellowship (Bordeaux University) and Region Nouvelle-Aquitaine. We thank Dr. Samuel Weiss and Dr. Artee Luchman at the University of Calgary for sharing BTSC73.

## Author contributions

Performed the experiments, C.T., M.T., A.S., and A. Burban; designed the experiments and analyzed the data, C.T., M.T., A.S., and A. Burban; resources, T.D., A. Bikfalvi, L.B., T.M., and A.C.P.Z.; writing – original draft, C.T., M.T., A.S., and A. Burban; writing – review and editing, C.T., M.T., A. Bikfalvi, T.D., L.B., T.M., A.C.P.Z., A. Burban, and A.S.; conceived the research program and provided funding and mentorship, A. Burban, L.B., and A.S.

## Declaration of interests

The authors declare no competing interests.
